# Accelerating MCMC algorithms

**DOI:** 10.1002/wics.1435

**Published:** 2018-06-13

**Authors:** Christian P. Robert, Víctor Elvira, Nick Tawn, Changye Wu

**Affiliations:** ^1^ Université Paris Dauphine PSL Research University Paris France; ^2^ Department of Statistics University of Warwick Coventry UK; ^3^ IMT Lille Douai Douai France; ^4^ CRIStAL Lille France

**Keywords:** Bayesian analysis, computational statistics, convergence of algorithms, efficiency of algorithms, Hamiltonian Monte Carlo, Monte Carlo methods, Rao‐Blackwellisation, simulation, tempering

## Abstract

Markov chain Monte Carlo algorithms are used to simulate from complex statistical distributions by way of a local exploration of these distributions. This local feature avoids heavy requests on understanding the nature of the target, but it also potentially induces a lengthy exploration of this target, with a requirement on the number of simulations that grows with the dimension of the problem and with the complexity of the data behind it. Several techniques are available toward accelerating the convergence of these Monte Carlo algorithms, either at the exploration level (as in tempering, Hamiltonian Monte Carlo and partly deterministic methods) or at the exploitation level (with Rao–Blackwellization and scalable methods).

This article is categorized under:

Statistical and Graphical Methods of Data Analysis > Markov Chain Monte Carlo (MCMC)
Algorithms and Computational Methods > Algorithms
Statistical and Graphical Methods of Data Analysis > Monte Carlo Methods

## 1 INTRODUCTION

Markov chain Monte Carlo (MCMC) algorithms have been used for nearly 60 years and have become a reference method for analyzing Bayesian complex models in the early 1990s (Gelfand & Smith, 1990). The strength of this method is that it guarantees convergence to the quantity (or quantities) of interest with minimal requirements on the targeted distribution (also called *target*) behind such quantities. In that sense, MCMC algorithms are robust or universal, as opposed to the most standard Monte Carlo methods (e.g., Rubinstein, 1981; Robert & Casella, 2004) that require direct simulations from the target distribution. This robustness may, however, induce a slow convergence behavior in that the exploration of the relevant space—meaning the part of the space supporting the distribution that has a significant probability mass under that distribution—may take a long while, as the simulation usually proceeds by local jumps in the vicinity of the current position. In other words, MCMC–especially in its off‐the‐shelf versions like Gibbs sampling and Metropolis–Hastings (MH) algorithms—is very often myopic in that it provides a good illumination of a local area, while remaining unaware of the global support of the distribution. As with most other simulation methods, there always exist ways of creating highly convergent MCMC algorithms by taking further advantage of the structure of the target distribution. Here, we mostly limit ourselves to the realistic situation where the target density is only known as the output of a computer code or to a setting similarly limited in its information content.

The approaches to the acceleration of MCMC algorithms can be divided in several categories, from those which improve our knowledge about the target distribution, to those that modify the proposal in the algorithm, including those that exploit better the outcome of the original MCMC algorithm. The following sections provide more details about these directions and the solutions proposed in the literature.

### 1.1 What is MCMC and why does it need accelerating?

MCMC methods have a history (e.g., Cappé & Robert, 2000) that starts at approximately the same time as the Monte Carlo methods, in conjunction with the conception of the first computers. They have been devised to handle the simulation of complex target distributions, when complexity stems from the shape of the target density, the size of the associated data, the dimension of the object to be simulated, or from time requirements. For instance, the target density *π*(*θ*) may happen to be expressed in terms of multiple integrals that cannot be solved analytically,

$$\pi \left(\theta \right)=\int \omega \left(\theta ,\xi \right)d\xi$$

which requires the simulation of the entire vector (*θ*, *ξ*). In cases when *ξ* is of the same dimension as the data, as for instance in latent variable models, this significant increase in the dimension of the object to be simulated creates computational difficulties for standard Monte Carlo methods, from managing the new target *ω*(*θ*, *ξ*), to devise a new and efficient simulation algorithm. A MCMC algorithm allows for an alternative resolution of this computational challenge by simulating a Markov chain that explores the space of interest (and possibly supplementary spaces of auxiliary variables) without requiring a deep preliminary knowledge on the density *π*, besides the ability to compute *π*(*θ*_0_) for a given parameter value *θ*_0_ (if up to a normalizing constant) and possibly the gradient *∇* log *π*(*θ*_0_). The validation of the method (e.g., Robert & Casella, 2004) is that the Markov chain is *ergodic* (e.g., Meyn & Tweedie, 1993), namely that it converges in distribution to the distribution with density *π*, no matter where the Markov chain is started at time *t* = 0.

The Metropolis–Hastings algorithm is a generic illustration of this principle. The basic algorithm is constructed by choosing a *proposal*, that is, a conditional density *K*(*θ* ^*′*^|*θ*) (also known as a *Markov kernel*), the Markov chain ${\left\{\theta_{t}\right\}}_{t=1}^{\infty }$ being then derived by successive simulations of the transition.

$$\theta_{t+1}=\left\{\begin{array}{ll} \theta^{\prime }∼K\left(\theta^{\prime }|\theta_{t}\right)\; & \text{with probability}\;\left\{\frac{\pi \left(\theta^{\prime }\right)}{\pi \left(\theta_{t}\right)}\times \frac{K\left(\theta_{t}|\theta^{\prime }\right)}{K\left(\theta^{\prime }|\theta_{t}\right)}\right\}\wedge 1,\\ \theta_{t} & \text{otherwise}. \end{array}\right.$$

This acceptance–rejection feature of the algorithm makes it appropriate for targeting *π* as its stationary distribution if the resulting Markov chain ${\left\{\theta_{t}\right\}}_{t=1}^{\infty }$ is irreducible, that is, has a positive probability of visiting any region of the support of *π* in a finite number of iterations. (Stationarity can easily be shown, e.g., by using the so‐called *detailed balance property* that makes the chain time‐reversible; see, e.g., Robert & Casella, 2004.)

Considering the initial goal of simulating samples from the target distribution *π*, the performances of MCMC methods like the Metropolis–Hastings algorithm above often vary quite a lot, depending primarily on the correspondance between the proposal *K* and the target *π*. For instance, if *K*(*θ*|*θ*_*t*_) = *π*(*θ*), the Metropolis–Hastings algorithm reduces to i.i.d. sampling from the target, which is of course a formal option when i.i.d. sampling from *π* proves impossible to implement. Although there exist rare instances when the Markov chain ${\left\{\theta_{t}\right\}}_{t=1}^{\infty }$ leads to negative correlations between the successive terms of the chain, making it *more efficient* than regular i.i.d. sampling (Liu, Wong, & Kong, 1995), the most common occurrence is one of positive correlation between the simulated values (sometimes uniformly, see Liu, Wong, & Kong, 1994). This feature implies a reduced efficiency of the algorithm and hence requires a larger number of simulations to achieve the same precision as an approximation based on i.i.d. simulations (without accounting for differences in computing time). More generally, a MCMC algorithm may require a large number of iterations to escape the attraction of its starting point *θ*_0_ and to reach stationarity, to the extent that some versions of such algorithms fail to converge in the time available (i.e., in practice if not in theory).

It thus makes sense to seek ways of accelerating (a) the convergence of a given MCMC algorithm to its stationary distribution, (b) the convergence of a given MCMC estimate to its expectation, and/or (c) the exploration of a given MCMC algorithm of the support of the target distribution. Those goals are related but still distinct. For instance, a chain initialized by simulating from the target distribution may still fail to explore the whole support in an acceptable number of iterations. While there is not an optimal and universal solution to this issue, below we will discuss approaches that are as generic as possible, as opposed to artificial ones taking advantage of the mathematical structure of a specific target distribution. Ideally, we aim at covering realistic situations when the target density is only known (up to a constant or an additional completion step) as the output of an existing computer code. Pragmatically, we also cover here solutions that require more efforts and calibration steps when they apply to a wide enough class of problems.

### 1.2 Accelerating MCMC by exploiting the geometry of the target

While there is no end in trying to construct more efficient and faster MCMC algorithms, and while this (endless) goal needs to account for the cost of devising such alternatives under limited resources budgets, there exist several generic solutions such that a given target can first be explored in terms of the geometry (or topology) of the density before constructing the algorithm. Although this type of methods somehow takes us away from our original purpose which was to improve upon an existing algorithm, they still make sense within this survey in that they allow for almost automated implementations.

### 1.3 Hamiltonian Monte Carlo

From the point of view of this review, Hamiltonian (or hybrid) Monte Carlo (HMC) is an auxiliary variable technique that takes advantage of a continuous time Markov process to sample from the target *π*. This approach comes from physics (Duane, Kennedy, Pendleton, & Roweth, 1987) and was popularized in statistics by Neal (1999, 2011) and MacKay (2002). Given a target *π*(*θ*), where *θ* ∈ ℝ^*d*^, an artificial auxiliary variable *ϑ* ∈ ℝ^*d*^ is introduced along with a density *ϖ*(*ϑ*|*θ*) so that the joint distribution of (*θ*, *ϑ*) enjoys *π*(*θ*) as its marginal. While there is complete freedom in this representation, the HMC literature often calls *ϑ* the *momentum* of a particle located at *θ* by analogy with physics. Based on the representation of the joint distribution.

$$\omega \left(\theta ,\vartheta \right)=\pi \left(\theta \right)\varpi \left(\vartheta |\theta \right)\propto \exp\left\{-H\left(\theta ,\vartheta \right)\right\}$$

where *H*(·) is called the *Hamiltonian*, Hamiltonian Monte Carlo (HMC) is associated with the continuous time process (*θ*_*t*_, *ϑ*_*t*_) generated by the so‐called *Hamiltonian equations*.

$$\frac{d\theta_{t}}{dt}=\frac{\partial H}{\partial \vartheta }\left(\theta_{t},\vartheta_{t}\right)\;\frac{d\vartheta_{t}}{dt}=-\frac{\partial H}{\partial \theta }\left(\theta_{t},\vartheta_{t}\right)$$

which keep the Hamiltonian target stable over time, as.

$$\frac{dH\left(\theta_{t},\vartheta_{t}\right)}{dt}=\frac{\partial H}{\partial \vartheta }\left(\theta_{t},\vartheta_{t}\right)\frac{d\vartheta_{t}}{dt}+\frac{\partial H}{\partial \theta }\left(\theta_{t},\vartheta_{t}\right)\frac{d\theta_{t}}{dt}=0.$$

Obviously, the above continuous time Markov process is deterministic and only explores a given level set,

$$\left\{\left(\theta ,\vartheta \right):H\left(\theta ,\vartheta \right)=H\left(\theta_{0},\vartheta_{0}\right)\right\},$$

instead of the whole augmented state space ℝ^2*d*^, which induces an issue with irreducibility. An acceptable solution to this problem is to refresh the momentum, *ϑ*_*t*_ ∼ *ϖ*(*ϑ*|*θ*_*t*−_), at random times ${\left\{\tau_{n}\right\}}_{n=1}^{\infty }$, where *θ*_*t*−_ denotes the location of *θ* immediately prior to time *t*, and the random durations ${\left\{\tau_{n}-\tau_{n-1}\right\}}_{n=2}^{\infty }$ follow an exponential distribution. By construction, continuous‐time Hamiltonian Markov chain can be regarded as a specific piecewise deterministic Markov process using Hamiltonian dynamics (Davis, 1984, 1993; Bou‐Rabee et al., 2017) and our target, *π*, is the marginal of its associated invariant distribution.

Before moving to the practical implementation of the concept, let us point out that the free cog in the machinery is the conditional density *ϖ*(*ϑ*|*θ*), which is usually chosen as a Gaussian density with either a constant covariance matrix *M* corresponding to the target covariance or as a local curvature depending on *θ* in Riemannian HMC (Girolami & Calderhead, 2011). Betancourt (2017) argues in favor of these two cases against non‐Gaussian alternatives and Livingstone, Faulkner, and Roberts (2017) analyze how different choices of kinetic energy in HMC affect algorithm performances. For a fixed covariance matrix, the Hamiltonian equations become:

$$\frac{d\theta_{t}}{dt}=M^{-1}\vartheta_{t}\;\frac{d\vartheta_{t}}{dt}=\nabla L\left(\theta_{t}\right)$$

which is the score function. The velocity (or momentum) of the process is thus driven by this score function, gradient of the log‐target.

The above description remains quite conceptual in that there is no generic methodology for producing this continuous time process, since the Hamiltonian equations cannot be solved exactly in most cases. Furthermore, standard numerical solvers like Euler's method create an instable approximation that induces a bias as the process drifts away from its true trajectory. There exists, however, a discretization simulation technique that produces a Markov chain and is well‐suited to the Hamiltonian equations, in that it preserves the stationary distribution (Betancourt, 2017). It is called the *symplectic integrator*, and one version in the independent case with constant covariance consists in the following (so‐called *leapfrog*) steps

$$\begin{array}{ll} \vartheta_{t+ɛ/2} & =\vartheta_{t}+\mathrm{ɛ\nabla}L\left(\theta_{t}\right)/2,\\ \theta_{t+ɛ} & =\theta_{t}+ɛM^{-1}\vartheta_{t+ɛ/2},\\ \vartheta_{t+ɛ} & =\vartheta_{t+ɛ/2}+\mathrm{ɛ\nabla}L\left(\theta_{t+ɛ}\right)/2, \end{array}$$

where *ɛ* is the time‐discretization step. Using a proposal on ϑ_0_ drawn from the Gaussian auxiliary target and deciding on the acceptance of the value of (*θ*_*Tɛ*_, *ϑ*_*Tɛ*_) by a Metropolis–Hastings step can limit the danger of missing the target. Note that the first two leapfrog steps induce a Langevin move on *θ*_*t*_:

$$\theta_{t+ɛ}=\theta_{t}+{ɛ}^{2}M^{-1}\nabla L\left(\theta_{t}\right)/2+ɛM^{-1}\vartheta_{t}$$

thus connecting with the Metropolis‐adjusted Langevin algorithm (MALA) discussed below (see Durmus and Moulines, 2017 for a theoretical discussion of the optimal choice of *ɛ*). Note that the leapfrog integrator is quite an appealing middleground between accuracy (as it is second‐order accurate) and computational efficiency.

In practice, it is important to note that discretizing the Hamiltonian dynamics introduces two free parameters, the step size *ɛ* and the trajectory length *Tɛ*, both to be calibrated. As an empirically successful and popular variant of HMC, the “no‐U‐turn sampler” (NUTS) of Hoffman and Gelman (2014) adapts the value of *ɛ* based on primal‐dual averaging. It also eliminates the need to choose the trajectory length *T* via a recursive algorithm that builds a set of candidate proposals for a number of forward and backward leapfrog steps and stops automatically when the simulated path steps back.

A further acceleration step in this area is proposed by Rasmussen (2003) (see also Fielding, Nott, & Liong, 2011), namely the replacement of the exact target density *π*(·) by an approximation $\hat{\pi }\left(\cdot \right)$ that is much faster to compute in the many iterations of the HMC algorithm. A generic way of constructing this approximation is to rely on Gaussian processes, when interpreted as prior distributions on the target density *π*(·), which is only observed at some values of *θ*, *π*(*θ*_1_), …, *π*(*θ*_*n*_) (Rasmussen and Williams, 2005). This solution is speeding up the algorithm, possibly by orders of magnitude, but it introduces a further approximation into the Monte Carlo approach, even when the true target is used at the end of the leapfrog discretization, as in Fielding et al. (2011).

**Figure 1:**
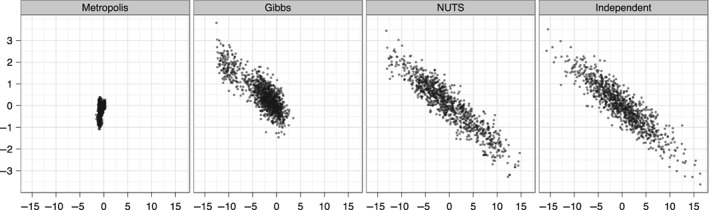
Comparisons between random‐walk Metropolis‐Hastings, Gibbs sampling, and NUTS algorithm of samples corresponding to a highly correlated 250‐dimensional multivariate Gaussian target. Similar computation budgets are used for all methods to produce the 1,000 samples on display. Source: Hoffman and Gelman (2014)

Stan (named after Stanislas Ullam, see Carpenter et al., 2017) is a computer language for Bayesian inference that, among other approximate techniques, implements the NUTS algorithm to remove hand‐tuning. More precisely, Stan is a probabilistic programming language in that the input is at the level of a statistical model, along with data, rather than the specifics of an MCMC algorithm. The algorithmic part is somehow automated, meaning that when models can be conveniently defined through this language, it offers an alternative to the sampler that produced the original chain. As an illustration of the acceleration brought by HMC, Figure 1, reproduced from Hoffman and Gelman (2014), shows the performance of NUTS, compared with both random‐walk MH and Gibbs samplers.

### 1.4 Accelerating MCMC by breaking the problem into pieces

The explosion in the collection and analysis of “big” data sets in recent years has brought new challenges to the MCMC algorithms that are used for Bayesian inference. When examining whether or not a new proposed sample is accepted at the accept–reject step, an MCMC algorithm such as the Metropolis–Hastings version needs to sweep over the whole data set, at each and every iteration, for the evaluation of the likelihood function. MCMC algorithms are then difficult to scale up, which strongly hinders their application in big data settings. In some cases, the data sets may be too large to fit on a single machine. It may also be that confidentiality measures impose different databases to stand on separate networks, with the possible added burden of encrypted data (Aslett, Esperança, & Holmes, 2015). Communication between the separate machines may prove impossible on an MCMC scale that involves thousands or hundreds of thousands of iterations.

### 1.5 Scalable MCMC methods

In the recent years, efforts have been made to design *scalable* algorithms, namely, solutions that manage to handle large‐scale targets by breaking the problem into manageable or scalable pieces. Roughly speaking, these methods can be classified into two categories (Bardenet, Doucet, & Holmes, 2015): divide‐and‐conquer approaches and subsampling approaches.

Divide‐and‐conquer approaches partition the whole data set, denoted X, into batches, $\left\{X_{1},\cdots ,X_{k}\right\}$, and run separate MCMC algorithms on each data batch, independently, as if they were independent Bayesian inference problems.^1^ These methods then combine the simulated parameter outcomes together to approximate the original posterior distribution. Depending on the treatments of the batches selected in the MCMC stages, these approaches can be further subdivided into two finer groups: subposterior methods and boosted subposterior methods. Subposterior methods are motivated by the independent product equation:

(1) $$\pi \left(\theta \right)\propto \prod_{i=1}^{k}\left(\pi_{0}{\left(\theta \right)}^{1/k}\prod_{\ell \in X_{i}}p(x_{\ell }|\theta )\right)=\prod_{i=1}^{k}\pi_{i}\left(\theta \right)$$

and they target the densities *π*_*i*_(*θ*) (up to a constant) in their respective MCMC steps. They thus bypass communication costs (Scott et al., 2016), by running MCMC samplers independently on each batch, and they most often increase MCMC mixing rates (in effective samples sizes produced by second), given that the subposterior distributions *π*_*i*_(*θ*) are based on smaller data sets. For instance, Scott et al. (2016) combine the samples from the subposteriors, *π*_*i*_(*θ*), by a Gaussian reweighting. Neiswanger, Wang, and Xing (2013) estimate the subposteriors *π*_*i*_(*θ*) by nonparametric and semi‐parametric methods, and they run additional MCMC samplers on the product of these estimators toward approximating the true posterior *π*(*θ*). Wang and Dunson (2013) refine this product estimator with an additional Weierstrass sampler, while Wang, Guo, Heller, and Dunson (2015) estimate the posterior by partitioning the space of samples with step functions.

As an alternative to sampling from the subposteriors, boosted subposterior methods target instead the components

(2) $${\tilde{\pi }}_{i}\left(\theta \right)\propto \pi_{0}\left(\theta \right){\left(\prod_{\ell \in X_{i}}p\left(x_{\ell }|\theta \right)\right)}^{k}$$

in separate MCMC runs. Since they formaly amount to repeating each batch *k* times toward producing pseudo data sets with the same size as the true one, the resulting boosted subposteriors, ${\tilde{\pi }}_{1}\left(\theta \right),\cdots ,{\tilde{\pi }}_{k}\left(\theta \right)$, have the same scale in variance of each component of the parameters, *θ*, as the true posterior, and can thus be treated as a group of estimators of the true posterior. In the subsequent combining stage, these subposteriors are merged together to construct a better approximation of the target distribution. For instance, Minsker, Srivastava, Lin, and Dunson (2014) approximate the posterior with the geometric median of the boosted subposteriors, embedding them into associated reproducing kernel Hilbert spaces, while Srivastava, Cevher, Dinh, and Dunson (2015) achieve this goal using the barycenters of ${\tilde{\pi }}_{1},\cdots ,{\tilde{\pi }}_{k}$, these barycenters being computed with respect to a Wasserstein distance.

In a perspective different from the above parallel scheme of divide‐and‐conquer approaches, subsampling approaches aim at reducing the number of individual datapoint likelihood evaluations operated at each iteration toward accelerating MCMC algorithms. From a general perspective, these approaches can be further classified into two finer classes: exact subsampling methods and approximate subsampling methods, depending on their resulting outputs. Exact subsampling approaches typically require subsets of data of random size at each iteration. One solution to this effect is taking advantage of pseudo‐marginal MCMC via constructing unbiased estimators of the target density evaluated on subsets of the data (Andrieu & Roberts, 2009). Quiroz, Villani, and Kohn (2016) follow this direction by combining the powerful debiasing technique of Rhee and Glynn (2015) and the correlated pseudo‐marginal MCMC approach of Deligiannidis, Doucet, and Pitt (2015). Another direction is to use piecewise deterministic Markov processes (PDMP) (Davis, 1984, 1993), which enjoy the target distribution as the marginal of their invariant distribution. This PDMP version requires unbiased estimators of the gradients of the log‐likelihood function, instead of the likelihood itself. By using a tight enough bound on the event rate function of the associated Poisson processes, PDMP can produce super‐efficient scalable MCMC algorithms. The bouncy particle sampler (Bouchard‐Côté, Vollmer, & Doucet, 2017) and the zig‐zag sampler (Bierkens, Fearnhead, & Roberts, 2016) are two competing PDMP algorithms, while Bierkens et al. (2017) unify and extend these two methods. Besides, one should note that PDMP produces a non‐reversible Markov chain, which means that the algorithm should be more efficient in terms of mixing rate and asymptotic variance, when compared with reversible MCMC algorithms, such as MH, HMC, and MALA, as observed in some theoretical and experimental works (Bierkens, 2016; Chen & Hwang, 2013; Hwang, Hwang‐Ma, & Sheu, 1993; Sun, Gomez, & Schmidhuber, 2010).

Approximate subsampling approaches aim at constructing an approximation of the target distribution. Beside the aforementioned attempts of Rasmussen (2003) and Fielding et al. (2011), one direction is to approximate the acceptance probability with high accuracy by using subsets of the data (Bardenet et al., 2015; Bardenet, Doucet, & Holmes, 2014). Another solution is based on a direct modification of exact methods. The seminal work of Welling and Teh (2011), stochastic gradient Langevin dynamics (SGLD), is to exploit the Langevin diffusion

(3) $$d\theta_{t}=\frac{1}{2}\Lambda \nabla \log\pi \left(\theta_{t}\right)dt+\Lambda^{1/2}dB_{t},\;\theta_{0}\in {\mathbb{R}}^{d},t\in \left[0,\infty \right)$$

where **Λ** is a user‐specified matrix, *π* is the target distribution, and **B**_*t*_ is a *d*‐dimensional Brownian process. By virtue of the Euler–Maruyama discretization and using unbiased estimators of the gradient of the log‐target density, SGLD and its variants (Chen, Fox, & Guestrin, 2014; Ding et al., 2014) often produce fast and accurate results in practice when compared with MCMC algorithms using MH steps.

**Figure 2:**
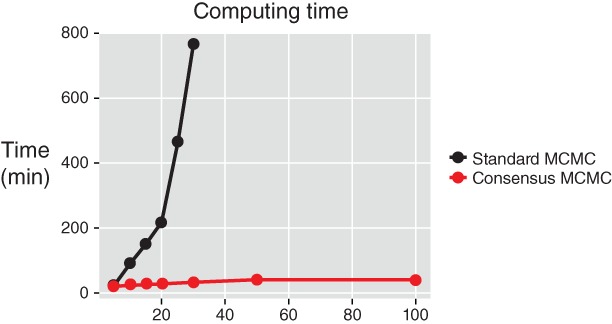
Elapsed time when drawing 10,000 MCMC samples with different amounts of data under the single machine and consensus Monte Carlo algorithms for a hierarchical Poisson regression. The horizontal axis represents the amounts of data. The single machine algorithm stops after 30 because of the explosion in computation budget. Source: Scott et al. (2016)

**Figure 3:**
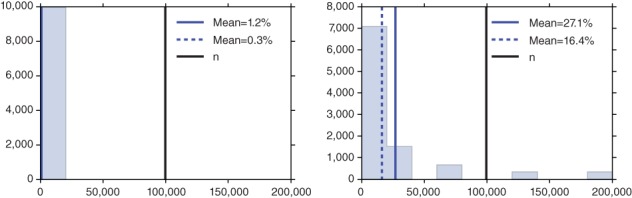
Percentage of numbers of data points used in each iteration of the confidence sampler with a single 2nd‐order Taylor approximation at *θ*_MAP_. The plots describe 10,000 iterations of the confidence sampler for the posterior distribution of the mean and variance of a unidimensional normal distribution with a flat prior: (left) 10,000 observations are generated from $N\left(0,\;,1\right)$, (right) 10,000 observations are generated from $LN\left(0,\;,1\right)$_._ Source: Bardenet et al. (2015)

Figure 2 shows the time requirements of a consensus Monte Carlo algorithm (Scott et al., 2016) compared with a Metropolis–Hastings algorithm using the whole data set, while Figure 3 displays the saving in likelihood evaluations in confidence sampler of Bardenet et al. (2015).

### 1.6 Parallelization and distributed schemes

Modern computational architectures are built with several computing units that allow for parallel processing, either fully independent or with certain communication. Although the Markovian nature of MCMC is inherently sequential and somewhat alien to the notion of parallelizing, several partial solutions have been proposed in the literature for exploiting these parallel architectures. The simplest approach consists in running several MCMC chains in parallel, blind to all others, until the allotted computing time is exhausted. Finally, the resulting estimators of all chains are averaged. However, this naive implementation may suffer from the fact that some of those chains have not reached their stationary regime by the end of the computation time, which then induces a bias in the resulting estimate. Ensuring that stationarity has been achieved is a difficult (if at all possible) task, although several approaches can be found in the literature (Guihenneuc‐Jouyaux & Robert, 1998; Jacob, O'Leary, & Atchadé, 2017; Mykland, Tierney, & Yu, 1995). At the opposite extreme, complex targets may be represented as products that involve many terms that must be evaluated, each of which can be attributed to a different thread before being multiplied all together. This strategy requires communication among processors at each MCMC step. A middle‐ground version (Jacob, Robert, & Smith, 2011) consists in running several Markov chains in parallel with periodic choices of the reference chain, all simulations being recycled through a Rao–Blackwell scheme. (See also Calderhead, 2014 for a similar scheme.) The family of interacting *orthogonal* MCMC methods (O‐MCMC) is proposed in Martino, Elvira, Luengo, Corander, and Louzada (2016) with the aim of fostering better exploration of the state space, specially in high‐dimensional and multimodal targets. Multiple MCMC chains are run in parallel exploring the space with random‐walk proposals. The parallel chains periodically share information, also through joint MCMC steps, thus allowing an efficient combination of global (coordinated) exploration and local approximation. O‐MCMC methods also allow for a parallel implementation of the Multiple Try Metropolis. In Calderhead (2014), a generalization of the Metropolis‐Hastings algorithm allows for a straightforward parallelization. Each proposed point can be evaluated in a different processor at every MCMC iteration. Finally, note that the section on scalable MCMC also contains parallelizable approaches, such as the prefetching method of Angelino, Kohler, Waterland, Seltzer, and Adams (2014) (see also Banterle, Grazian, Lee, & Robert, 2015 for a related approach, primarily based on an approximation of the target). A most recent endeavor called asynchronous MCMC (Terenin, Simpson, & Draper, 2015) aims at higher gains in parallelization by reducing the amount of exchange between the parallel threads, but the notion still remains confidential at this stage.

### 1.7 Accelerating MCMC by improving the proposal

In the same spirit as the previous section, this section is stretching the purpose of this paper by considering possible modifications of the MCMC algorithm itself, rather than merely exploiting the output of a given MCMC algorithm. For instance, devising an HMC algorithm is an answer to this question even though the “improvement” is not garanteed. Nonetheless, our argument here is that, once provided with this output, it is possible to derive new proposals in a semi‐autonomous manner.

### 1.8 Simulated tempering

The target distribution, *π*(*θ*) on *d*‐dimensional state space Θ, can exhibit multimodality with the probability mass being located in different regions in the state space. The majority of MCMC algorithms use a localized proposal mechanism which is tuned toward local approximate optimality see, for example, Roberts, Gelman, and Gilks (1997) and Roberts and Rosenthal (2001). By construction, these localized proposals result in the Markov chain becoming “trapped” in a subset of the state space meaning that in finite run‐time the chain can entirely fail to explore other modes in the state space, leading to biased samples. Strategies to accelerate MCMC often use local gradient information and this draws the chain back toward the center of the mode, which is the opposite of what is required in a multimodal setting.

There is an array of methodology available to overcome issues of multimodality in MCMC, the majority of which use state space augmentation. Auxiliary distributions that allow a Markov chain to explore the entirety of the state space are targeted and their mixing information is then passed on to aid mixing in the true target. While the subposteriors of the previous section can be seen as special cases of the following, the most successful and convenient implementation of these methods is to use *power‐tempered target distributions*. The target distribution at inverse temperature level, *β*, for *β* ∈ (0, 1] is defined as

$$\pi_{\beta }\left(\theta \right)=K\left(\beta \right){\left[\pi \left(\theta \right)\right]}^{\beta }\;\text{where}\;K\left(\beta \right)={\left[\int {\left[\pi \left(\theta \right)\right]}^{\beta }\mathrm{d\theta}\right]}^{-1}.$$

Therefore, *π*_1_(*θ*) = *π*(*θ*). Temperatures *β* < 1 flatten out the target distribution allowing the chain to explore the entire state space provided the *β* value is sufficiently small. The simulated tempering (ST) and parallel tempering (PT) algorithms (Geyer, 1991; Marinari & Parisi, 1992) typically use the power‐tempered targets to overcome the issue of multimodality. The ST approach runs a single Markov chain on the augmented state space {*B*, Θ}, where *B* = {*β*_0_, *β*_1_, …, *β*_*n*_} is a discrete collection of *n* inverse temperature levels with 1 = *β*_0_ > *β*_1_ > … > *β*_*n*_ > 0. The algorithm uses a Metropolis‐within‐Gibbs strategy by cycling between updates in the Θ and *B* components of the space. For instance, a proposed temperature swap move *β*_*i*_ → *β*_*j*_ is accepted with probability

$$\min\left\{1,\frac{\pi_{\beta_{j}}\left(\theta \right)}{\pi_{\beta_{i}}\left(\theta \right)}\right\}$$

in order to preserve detailed balance. Note that this acceptance ratio depends on the normalization constants $K\left(\beta \right)$ which are typically unknown, although they can sometimes be estimated, as in, for example, Wang and Landau (2001) and Atchadé and Liu (2004). In case estimation of the marginal normalization constants is impractical then the PT algorithm is employed. This approach simultaneously runs a Markov chain at each of the *n* + 1 temperature levels targeting the joint distribution given by $\prod_{i=0}^{n}{\left[\pi \left(\theta_{i}\right)\right]}^{\mathrm{\beta i}}$. Swap moves between chains at adjacent temperature levels are accepted according to a ratio that no longer depends on the marginal normalization constants. Indeed, this power tempering approach has been successfully employed in a number of settings and is widely used for example, Neal (1996), Earl and Deem (2005), Xie, Zhou, and Jiang (2010), Mohamed, Calderhead, Filippone, Christie, and Girolami, (2012) and Carter and White (2013).

In both approaches, there is a “Goldilocks” principle to setting up the inverse temperature schedule. Spacings between temperature levels that are “too large” result in swap moves that are rarely accepted, hence delaying the transfer of hot state mixing information to the cold states. On the other hand, spacings that are too small require a large number of intermediate temperature levels, again resulting in slow mixing through the temperature space. This problem becomes even more difficult as the dimensionality of Θ increases.

Much of the historical literature suggested that a geometric spacing was optimal that is, there exists *c* ∈ (0, 1) such that *β*_*i* + 1_ = *cβ*_*i*_ for *i* = 0, 1, …, *n*. However, in the case of the ST version, Atchadé, Roberts, and Rosenthal (2011) considered the problem as an optimal scaling problem by maximizing the (asymptotic in dimension) expected squared jumping distance in the *B* space for temperature swap moves. Under restrictive assumptions, they showed that the spacings between consecutive inverse temperature levels should scale with dimension as *O*(*d*^−1/2^) to prevent degeneracy of the swap move acceptance rate. For a practitioner the result gave guidance on optimal setup since it suggested a corresponding optimal swap move acceptance rate of 0.234 between consecutive inverse temperature levels, in accordance with Gelman, Gilks, and Roberts (1996). Finally, contrary to the historically recommended geometric schedule, the authors suggested that temperature schedule setup should be constructed consecutively so as to induce an approximate 0.234 swap acceptance rate between consecutive levels; which is achieved adaptively in Miasojedow, Moulines, and Vihola (2013). The use of expected squared jumping distance as the measure of mixing speed was justified in Roberts and Rosenthal (2014) where, under the same conditions as in Atchadé et al. (2011), it was shown that the temperature component of the ST chain has an associated diffusion process.

**Figure 4:**
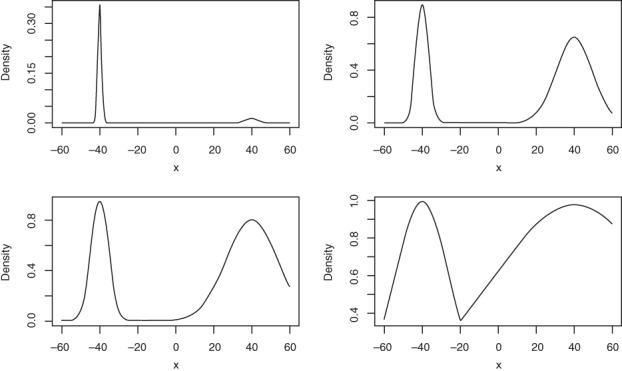
Unnormalized tempered target densities of a bimodal Gaussian mixture using inverse temperature levels *β* = {1, .1, .05, .005}, respectively. At the hot state (bottom right) it is evident that the mode centred on 40 begins to dominate the weight as *β* increases to ∞ even though at the cold state it was only attributable for a fraction (.2) of the total mass

The target of an 0.234 acceptance rate gives good guidance to setting up the ST/PT algorithms in certain settings, but there is a major warning for practitioners following this rule for optimal setup. The assumptions made in Atchadé et al. (2011) and Roberts and Rosenthal (2014) ignore the restrictions of mixing within a temperature level, instead assuming that this can be done infinitely fast relative to the mixing within the temperature space. Woodard, Schmidler, and Huber (2009a, 2009b)and Bhatnagar and Randall (2016) undertake a comprehensive analysis of the spectral gap of the ST/PT chains and their conclusion is rather damning of the ST/PT approaches that use power‐tempered targets. Essentially, in situations where the modes have different structures, the time required to reach a given level of convergence for the ST/PT algorithms can grow exponentially in dimension. A major reason for this is that power‐based tempering does not preserve the relative weights/mass between regions at the different temperature levels, see Figure 4. This issue can scale exponentially in dimension. From a practical perspective, in these finite run high‐dimensional nonidentical modal structure settings the swap acceptance rates can be very misleading, meaning that they have limited use as a diagnostic for intermodal mixing quality.

### 1.9 Adaptive MCMC

Improving and calibrating an MCMC algorithm toward a better correspondance with the intended target is a natural step in making the algorithm more efficient, provided enough information is available about this target distribution. For instance, when an MCMC sample associated with this target is available, even when it has not fully explored the range of the target, it contains some amount of information, which can then be exploited to construct new MCMC algorithms. Some of the solutions available in the literature (e.g., Liang, Liu, & Carroll, 2007) proceed by repeating blocks of MCMC iterations and updating the proposal *K* after each block, aiming at a particular optimality goal like a specific acceptance rate like 0.234 for Metropolis–Hastings steps (Gelman et al., 1996). Most versions of this method update the scale structure of a random walk proposal, based on previous realizations (Robert & Casella, 2009) or on an entire sample (Douc, Guillin, Marin, & Robert, 2007), which turns the method into iterated importance sampling with Markovian dependence. (It can also be seen as a static version of particle filtering, Doucet, Godsill, & Andrieu, 2000; Andrieu & Doucet, 2002; Storvik, 2002.)

**Figure 5:**
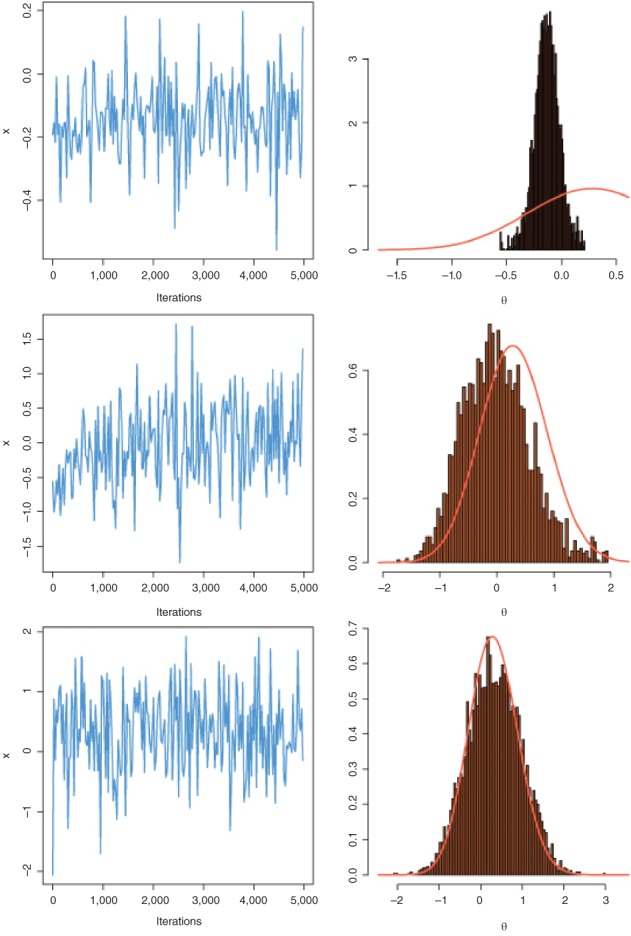
Markov chains produced by an adaptive algorithm where the proposal distribution is a Gaussian distribution with mean and variance computed from the past simulations of the chain. The three rows correspond to different initial distributions. The fit of the histogram of the resulting MCMC sample is poor, even for the most spread‐out initial distribution (bottom). Source: Robert and Casella (2004)

Other adaptive resolutions bypass this preliminary and somewhat ad hoc construction and aim instead at a permanent updating within the algorithm, motivated by the idea that a continuous adaptation keeps improving the correspondance with the target. In order to preserve the validation of the method (Gelman et al., 1996; Haario, Saksman, & Tamminen, 1999; Roberts & Rosenthal, 2007; Saksman & Vihola, 2010), namely that the chain produced by the algorithm converges to the intended target, specific convergence results need be established, as the ergodic theorem behind standard MCMC algorithms does not apply. Without due caution (see Figure 5), an adaptive MCMC algorithm may fail to converge due to over‐fitting. A drawback of adaptivity is that the update of the proposal distribution relies *too much* on the earlier simulations and thus reinforces the exclusion of parts of the space that have not yet been explored.

For the validation of adaptive MCMC methods, stricter constraints must thus be imposed on the algorithm. One well‐described solution (Roberts & Rosenthal, 2009) is called *diminishing adaptation*. Informally, it consists in imposing a distance between two consecutive proposal kernels to uniformly decrease to zero. In practice, this means stabilizing the changes in the proposal by ridge‐like factors as in the early proposal by Haario et al. (1999). A drawback of this resolution is that the decrease itself must be calibrated and may well fail to bring a significant improvement over the original proposal.

### 1.10 Multiple try MCMC

A completely different approach to improve the original proposal used in an MCMC algorithm is to consider a collection of proposals, built on different rationales and experiments. The *multiple try MCMC algorithm* (Liu, Liang, & Wong, 2000; Bédard, Douc, & Moulines, 2012; Martino, 2018) follows this perspective. As the name suggests, the starting point of a multiple try MCMC algorithm is to simultaneously propose *N* potential moves $\theta_{t}^{1},\ldots ,\theta_{t}^{N}$ of the Markov chain, instead of a single value. The proposed values $\theta_{t}^{i}$ may be independently generated according to *N* different proposal densities *K*_*i*_(·|*θ*_*t*_) that are conditional on the current value of the Markov chain, *θ*_*t*_. One of the $\theta_{t}^{i}$’s is selected based on the importance sampling weights $w_{t}^{i}\propto \pi \left(\theta_{t}^{i}\right)/K_{i}\left(\cdot |\theta_{t}\right)$. The selected value is then accepted by a further Metropolis–Hastings step which involves a ratio of normalization constants for the importance stage, one corresponding to the selection made previously and another one created for this purpose. Indeed, besides the added cost of computing the sum of the importance weights and generating the different variates, this method faces the non‐negligible drawback of requiring *N* − 1 supplementary simulations that are only used for achieving detailed balance and computing a backward summation of importance weights. This constraint may vanish when considering a collection of independent Metropolis‐Hastings proposals, *q*(*θ*), but this setting is rarely realistic as it requires some amount of prior knowledge or experimentation to build a relevant distribution.

**Figure 6:**
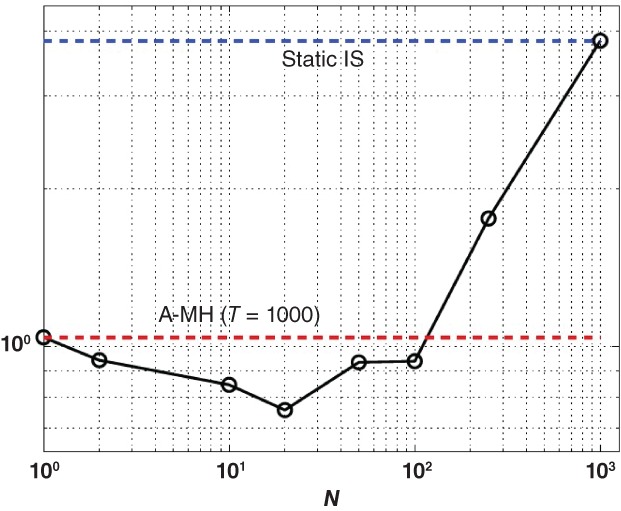
A comparison of an ensemble MCMC approach with a regular adaptive MCMC algorithm (lower line) and a static importance sampling approach, in terms of mean square error (MSE), for a fixed total number of likelihood evaluations, where *N* denotes the size of the ensemble. Source: Martino (2018)

An alternative found in the literature is *ensemble Monte Carlo* (Iba, 2000; Cappé, Douc, Guillin, Marin, & Robert, 2008; Neal, 2011; Martino, 2018), illustrated in Figure 6 which produces a whole sample at each iteration, with target the product of the initial targets, in closer proximity with particle methods (Cappé, Guillin, Marin, & Robert, 2004; Mengersen & Robert, 2003).

Yet another implementation of this principle is called *delayed rejection* (Tierney & Mira, 1998; Mira, 2001; Mira & Sargent, 2003), where proposals are instead considered sequentially, once the previous proposed value has been rejected, to speed up MCMC by considering several possibilities, if sequentially. A computational difficulty with this approach is that the associated acceptance probabilities get increasingly complex as the number of delays grows, which may annihilate its appeal relative to simultaneous multiple tries. A further difficulty is to devise the sequence of proposals in a diverse enough manner.

### 1.11 Accelerating MCMC by reducing the variance

Since the main goal of MCMC is to produce approximations for quantities of interest of the form,

$$I_{h}=\int_{\Theta }h\left(\theta \right)\pi \left(\theta \right)d\theta ,$$

an alternative (and cumulative) way of accelerating these algorithms is to improve the quality of the approximation derived from an MCMC output. That is, given an MCMC sequence *θ*_1_, …, *θ*_*T*_, converging to *π*(·), one can go beyond resorting to the basic Monte Carlo approximation

(4) $${\hat{I}}_{h}^{T}=\frac{1}{T}\sum_{t=1}^{T}h\left(\theta_{t}\right)$$

toward reducing the variance (if not the speed of convergence) of ${\hat{I}}_{h}^{T}$ to I_*h*_.

A common remark when considering Monte Carlo approximations of I_*h*_ is that the representation of the integral as an expectation is not unique (e.g., Robert & Casella, 2004). This leads to the technique of importance sampling where alternative distributions are used in replacement of *π*(*θ*), possibly in an adaptive manner (Douc et al., 2007), or sequentially as in particle filters (Andrieu, Doucet, & Holenstein, 2011; Del Moral, Doucet, & Jasra, 2006). Within the framework of this essay, the outcome of a given MCMC sampler can also be exploited in several ways that lead to an improvement of the approximation of I_*h*_.

### 1.12 Rao–Blackwellization and other averaging techniques

The name “Rao–Blackwellisation” was coined by Gelfand and Smith (1990) in their foundational Gibbs sampling paper and it has since then become a standard way of reducing the variance of integral approximations. While it essentially proceeds from the basic probability identity.

$$E^{\pi }\left[h\left(\theta \right)\right]=E^{\pi_{1}}\left[E^{\pi_{2}}\left\{h\left(\theta \right)|\xi \right\}\right],$$

when *π* can be expressed as the following marginal density

$$\pi \left(\theta \right)=\int_{\Xi }\pi_{1}\left(\xi \right)\pi_{2}\left(\theta |\xi \right)d\xi ,$$

and while sufficiency does not have a clear equivalence for Monte Carlo approximation, the name stems from the Rao–Blackwell theorem (Lehmann & Casella, 1998) that improves upon a given estimator by conditioning upon a sufficient statistics. In a Monte Carlo setting, this means that Equation (4) can be improved by a partly integrated version

(5) $${\tilde{I}}_{h}^{T}=\frac{1}{T}\sum_{t=1}^{T}E^{\pi_{2}}\left[h\left(\theta \right)|\xi^{t}\right]$$

assuming that a second and connected sequence of simulations (*ξ*_*t*_) is available and that the conditional expectation is easily constructed. For instance, Gibbs sampling (Gelfand & Smith, 1990) is often open to this Rao–Blackwell decomposition as it relies on successive simulations from several conditional distributions, possibly including auxiliary variates and nuisance parameters. In particular, a generic form of Gibbs sampling called the slice sampler (Robert & Casella, 2004) produces one or several uniform variates at each iteration.

However, a more universal type of Rao–Blackwellization is available (Casella & Robert, 1996) for all MCMC methods involving rejection, first and foremost, Metropolis–Hastings algorithms. Indeed, first, the distribution of the rejected variables can be derived or approximated, which leads to an importance correction of the original estimator. Furthermore, the accept–reject step depends on a uniform variate, but this uniform variate can be integrated out. Namely, given a sample produced by a Metropolis–Hastings algorithm *θ* ^(1)^, …, *θ* ^(*T*)^, one can exploit both underlying samples, the proposed values *ϑ*_1_, …, *ϑ*_*T*_, and the uniform *u*_1_, …, *u*_*T*_, so that the ergodic mean can be rewritten as

$${\hat{ℑ}}_{h}^{T}=\frac{1}{T}\sum_{t=1}^{T}h\left(\theta^{\left(t\right)}\right)=\frac{1}{T}\sum_{t=1}^{T}h\left(\vartheta_{t}\right)\sum_{i=t}^{T}I_{\theta^{\left(i\right)}=\vartheta_{t}}.$$

The conditional expectation

$$\begin{array}{ll} {\tilde{ℑ}}_{h}^{T} & =\frac{1}{T}\sum_{t=1}^{T}h\left(\vartheta_{t}\right)E\left[\sum_{i=t}^{T}I_{\theta^{\left(i\right)}=\vartheta_{t}}\left|\vartheta_{1},\ldots ,\vartheta_{T}\right.\right]\\ & =\frac{1}{T}\sum_{t=1}^{T}h\left(\vartheta_{t}\right)\left\{\sum_{i=t}^{T}\mathbb{P}\left(\theta^{\left(i\right)}=\vartheta_{t}|\vartheta_{1},\ldots ,\vartheta_{T}\right)\right\} \end{array}$$

then enjoys a smaller variance. See also Tjelmeland (2004) and Douc and Robert (2010) for connected improvements based on multiple tries. An even more rudimentary (and cheaper) version can be considered by integrating out the decision step at each Metropolis–Hastings iteration: if *θ*_*t*_ is the current value of the Markov chain and *ϑ*_*t*_ the proposed value, to be accepted (as *θ*_*t* + 1_) with probability *α*_*t*_, the version

$$\frac{1}{T}\sum_{t=1}^{T}\left\{\alpha_{t}h\left(\vartheta_{t}\right)+\left(1-\alpha_{t}\right)h\left(\theta_{t}\right)\right\}$$

should most often^2^ bring an improvement over the basic estimate (Liu et al., 1995; Robert & Casella, 2004).

## 2 CONCLUSIONS

Accelerating MCMC algorithms may sound like a new Achille versus tortoise paradox in that there are aways methods to speed up a given algorithm. The stopping rule of this infinite regress is, however, that the added pain in achieving this acceleration may overcome the added gain at some point. While we have only and mostly superficially covered some of the possible directions in this survey, we thus encourage most warmly readers to keep an awareness for the potential brought by a wide array of almost cost‐free accelerating solutions as well as to keep trying devising more fine‐tuned improvements in every new MCMC implementation. For instance, for at least one of us, Rao–Blackwellization is always considered at this stage. Keeping at least one such bag of tricks at one's disposal is thus strongly advised.

## CONFLICT OF INTEREST

The authors have declared no conflicts of interest for this article.

## RELATED WIREs ARTICLES

https://doi.org/10.1007/s11222-015-9574-5

## NOTES

1 In order to keep the notations consistent, we still denote the target density by *π*, with the prior density denoted as *π*_0_ and the sampling distribution of one observation *x* as *p*(*x*|*θ*). The dependence on the sample X is not reported unless necessary.

2 The improvement is not universal, due to the correlation between the terms of the sum induced by the Markovian nature of the sequence ${\left\{\theta_{t}\right\}}_{t=1}^{T}$.
